# ‘Top-Down’ holmium laser enucleation of the prostate. Report of initial cases performed by a single surgeon

**DOI:** 10.1080/2090598X.2020.1805964

**Published:** 2020-08-16

**Authors:** Amr Hodhod, Fabiola Oquendo, Thomas Tablowski, Ruba Abdul-Hadi, Walid Shahrour, Ahmed Kotb, Owen Prowse, Hazem Elmansy

**Affiliations:** Department of Urology, Northern Ontario School of Medicine, Thunder Bay, Ontario, Canada

**Keywords:** Benign prostatic hyperplasia, holmium laser, enucleation

## Abstract

**Objectives:**

To present the 12-month outcomes of ‘Top-Down’ holmium laser enucleation of the prostate (HoLEP).

**Patients and methods:**

We retrospectively reviewed the charts of prospectively collected patients who underwent Top-Down HoLEP between 2017 and 2018. All cases were operated upon by a single urologist (H.E), using a 100-W holmium:YAG laser with a 550-μm laser fibre. We recorded the enucleation time, morcellation time, intraoperative, and postoperative complications. All patients had postoperative follow-up visits at 1, 3, 6 and 12 months. The evaluation included the International Prostate Symptom Score (IPSS), quality-of-life (QoL) assessment, measurement of maximum urinary flow rate (Q_max_) and the post-void residual urine volume (PVR).

**Results:**

A total of 60 consecutive patients were recruited. The median (range) prostatic volume, resected prostatic weight, and percentage of resected prostatic tissue were 124 (70–266) mL, 90 (44–242) g and 76 (46–97)%, respectively. The median (range) enucleation and morcellation times were 80 (25–200) and 14.5 (4–58) min, respectively. One patient had a simple bladder mucosal injury and another developed clot retention. At 3 months, three patients (5%) had stress urinary incontinence (SUI) and eight patients (13.3%) presented with urge UI (UUI). At the last follow-up visit, one patient (1.7%) presented with persistent SUI, while three patients (5%) presented with UUI. The IPSS and QoL significantly improved during the follow-up period (*P* = 0.045 and *P* = 0.04, respectively).

**Conclusion:**

The results of the Top-Down technique are comparable to those of traditional HoLEP. However, the Top-Down technique may reduce the complexity, operating time, and SUI rates.

**Abbreviations:**

BN: bladder neck; HoLEP: holmium laser enucleation of the prostate; PVR: post-void residual urine volume; Q_max_: maximum urinary flow rate; QoL: quality of life; TOV: trial of voiding; (S)(U)UI: (stress) (urge) urinary incontinence

## Introduction

There has been a growing interest in replacing conventional surgical procedures, such as TURP and open prostatectomy, with holmium laser enucleation of the prostate (HoLEP) as the new standard for the surgical management of BPH for prostate glands of any size [1]. When compared to other surgical treatments of BPH, HoLEP has better long-term outcomes, fewer complications, and lower re-operation rates [2–4]. The major disadvantage of HoLEP, in comparison with standard TURP, is the longer duration of training required to master the procedure. HoLEP is considered a challenging surgery to learn. On average, 20 cases are required to achieve basic competency for performing HoLEP [5,6].

To simplify the procedure, some modifications were recently made to the traditional HoLEP technique, without altering the main concept or acceptable outcomes [7–9]. The ‘Top-Down’ technique is a novel anteroposterior HoLEP dissection procedure that was initially described by York et al. [10] in 2017. In the present study, we present our initial experience with Top-Down HoLEP.

## Patients and methods

We retrospectively reviewed the charts of prospectively collected patients who underwent Top-Down HoLEP between October 2017 and September 2018. All cases were operated on by a single urologist (H.E) who is a HoLEP expert (>300 cases). We included patients who presented with refractory urinary retention, refractory haematuria due to prostate enlargement, upper system affection, bladder stone secondary to BPH, and severe lower urinary tract obstruction that did not respond to medical treatment.

We used a 100-W holmium:YAG laser (VersaPulse® PowerSuite™, Lumenis, Yokneam, Israel) with a 550-μm laser fibre and a 28-F continuous flow resectoscope (Karl Storz SE & Co. KG, Tuttlingen, Germany). Enucleated tissue was morcellated using a Karl Storz DrillCut™ morcellator.

The preoperative data collected included: the patients’ presenting symptoms, a preoperative IPSS, post-void residual urine volume (PVR), urine test results, and the measurement of maximum urinary flow rate (Q_max_). A preoperative biopsy was taken in patients with PSA values above normal and/or abnormal DRE findings to exclude prostate cancer. Cystoscopy was preoperatively performed in patients who underwent previous TURP to exclude bladder neck (BN) contracture and/or urethral strictures. The technique of the Top-Down HoLEP is similar to that described previously [11].

**Figure 1.**
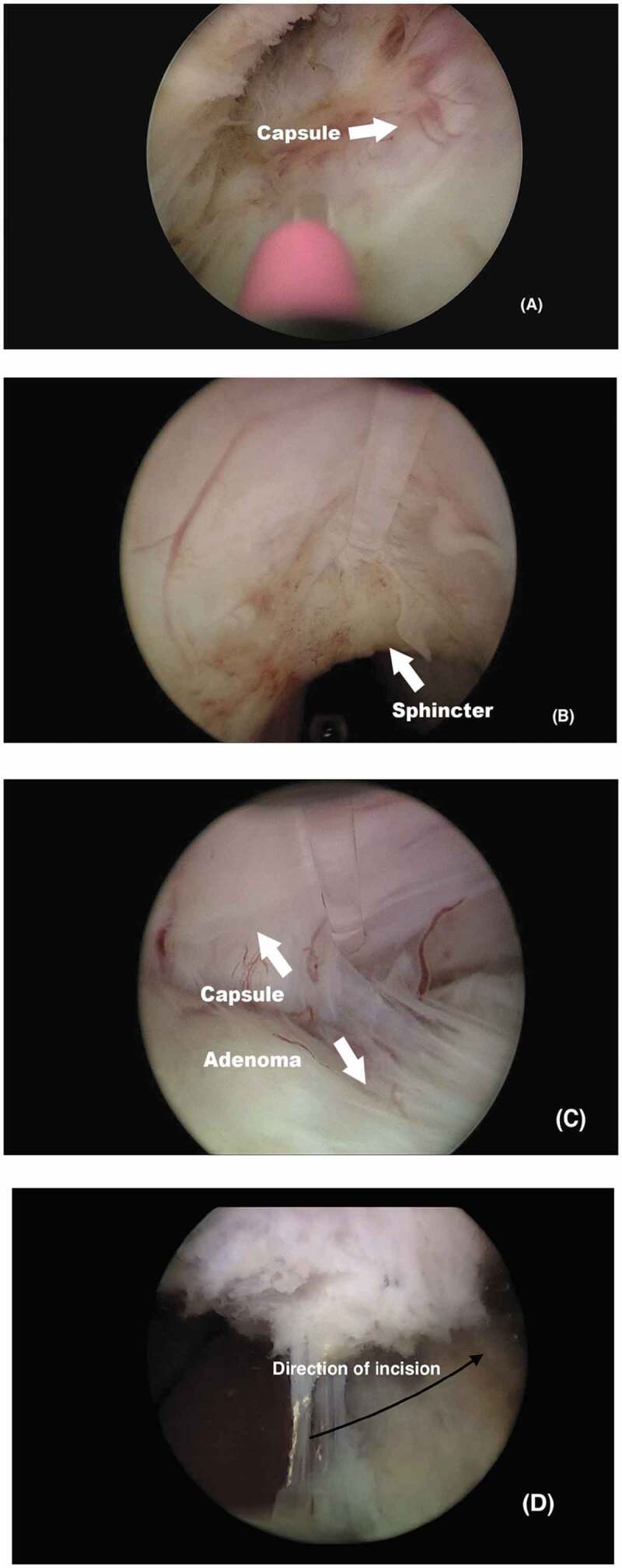
Important steps of Top-Down HoLEP

Firstly, one posterior groove was created at either the 5 or 7 o’clock position up to the verumontanum (Figure 1(a)), allowing simultaneous enucleation of the median lobe with the attached lateral lobe. Afterwards, the anterior commissure mucosa was then incised at 2 J/20 Hz starting from the BN at the 12 o’clock position (Figure 1(b)). The incision was carefully completed proximal to the external sphincter, then deepened to separate the area between the right and left adenoma, until reaching the surgical capsule. Once the plane between the adenoma and surgical capsule was created, a top-down lateral lobe dissection was performed and extended anteroposteriorly towards the apical adenoma at the 6 o’clock position (Figure 1(c)). The energy setting for dissection was 2 J/40 Hz. Once the surgeon reached the BN at the 6 o’clock position, the remaining attachment between the adenoma and surgical capsule was cautiously separated to avoid injuring the ureteric orifices at the BN from lateral to medial (Figure 1(d)). At that time, the adenoma was released and freely fell in the bladder. The other lateral lobe (with or without the attached median lobe) was enucleated in the same fashion. After morcellation, a 22-F three-way urethral catheter was inserted.

We recorded the surgical parameters, including enucleation time, enucleation efficiency, morcellation time, laser energy, and intraoperative complications. Intraoperative complications comprised intraoperative bleeding, bladder mucosal injury, and capsular perforation. Early postoperative complications included clot retention and failed trial of voiding (TOV). Late postoperative complications included stress urinary incontinence (SUI), urge UI (UUI), urethral strictures, and BN contraction. SUI was evaluated through a detailed history regarding the involuntary passage of urine while coughing or sneezing, or the use of pads to avoid wetting. Moreover, SUI was clinically evaluated by asking the patient, with a full bladder, to cough, and the passage of any urine was observed. UUI was considered if the patient had a sudden uninhibited desire to micturate with the passage of urine before reaching the toilet.

All patients had postoperative follow-ups at 1, 3, 6 and 12 months. Our evaluation included the IPSS, quality-of-life (QoL) assessment, Q_max_, and PVR. The PSA level measurement was conducted at 3 months. The percentage change of these outcome measures was calculated using the following formula: [(preoperative measure – postoperative measure)/(preoperative measure)] × 100. Our primary outcome was to evaluate the feasibility of Top-Down HoLEP by evaluating all postoperative measures at the four follow-up intervals. Our secondary outcome was to study the learning curve of the Top-Down approach. In order to assess the learning curve, we divided our patients chronologically into three groups (20 patients each). Thereafter, we compared the intraoperative data and postoperative outcomes of the three groups.

Data collection and statistical analysis were conducted using the Statistical Package for the Social Sciences (SPSS®), version 20 (SPSS Inc., IBM Corp., Armonk, NY, USA).

The Friedman two-way analysis test and Wilcoxon signed-rank tests were used to continuously evaluate data. The *P* value was considered significant if ≤0.05.

## Results

A total of 60 consecutive patients with BPH underwent Top-Down HoLEP; 94% (57/60) continued follow-up to 12 months. Patient demographics and preoperative data are listed in Table 1. The median (range) prostatic volume, median resected prostatic weight, and percentage of resected prostatic tissue were 124 (70–266) mL, 90 (44–242) g, and 76 (46–97)%, respectively. The median (range) energy used was 177 (81.5–319) kJ. The median (range) enucleation and morcellation times were 80 (25–200) and 14.5 (4–58) min, respectively. The median enucleation efficiency was 0.9 g/min and the median (range) morcellation rate 6.4 (2.9–15) g/min.

**Table 1.** Patients’ characteristics and preoperative data

Characteristic		Value
Age, years, median (range)		72.8 (54–88)
Presentation, *n* (%)	LUTS	24 (40)
	Retention	20 (33.4)
	Retention + bladder stones	6 (10)
	Haematuria	4 (6.7)
	Clot retention	3 (5)
	Retention + ARF	2 (3.3)
	LUTS + haematuria	1 (1.6)
Prostate size, mL, median (range)		124 (70–266)
Prostate size >100 mL, *n* (%)		37 (61.7)
Preoperative PSA level, ng/ml, median (range)		6 (0.84–27.1)
Preoperative IPSS, median (range)		21 (10–34)
Preoperative QoL, median (range)		5 (2–6)
Preoperative PVR, mL, median (range)		400 (11–2600)
Preoperative Q_max_, mL/s, median (range)		8.4 (5.5–15)
Recurrent after TURP, *n* (%)		16 (26.7)

A total of 55 patients (91.7%) had an overnight hospital stay. Two patients (3.3%) were hospitalised for 48 h due to postoperative fever and postoperative tachycardia. Three patients (5%) had a successful catheter removal trial on the same day. All of them were successful and were discharged on the same operative day.

Catheter removal was successfully performed the following morning in 55 patients (91.7%). Two patients (3.3%) had failed TOV the next morning postoperatively. One of the two patients had a bladder mucosal injury, while the other patient had prolonged catheterisation due to retention. Both patients successfully voided 5 days after discharge.

Upon reviewing the pathology reports, six patients (10%) had an incidental discovery of prostate cancer. Three patients (5%) had a Gleason score of 3 + 3 and the remaining had a Gleason score of 3 + 4. Moreover, there was an incidental discovery of superficial urothelial carcinoma of the bladder in two patients (3.3%).

### Postoperative complications

None of the patients required a blood transfusion. There were no intraoperative complications recorded, with the exception of one patient with a simple bladder mucosal injury. Another patient (1.7%) developed clot retention a few days after the surgery, as a result of an early return to heavy manual work. The patient was readmitted for clot evacuation using a three-way catheter.

At the 1-month follow-up visit, three patients (5%) had SUI, while eight patients (13.3%) had UUI. Of the patients with SUI, two became fully content at their 3-month follow-up visit. Five of the patients with UUI had satisfactory continence at the 3-month follow-up visit. During the last follow-up visit, only one patient (1.7%) presented with persistent SUI and three (5%) experienced UUI. In addition, one patient presented with meatal stenosis.

### Top-Down outcomes

The reviewed outcome measures are presented in Table 2. The subjective and objective parameters all significantly improved immediately after surgery. When comparing Q_max_ at the four time-intervals, they showed no great difference (*P* = 0.17). However, the Q_max_ at 12 months was significantly higher than that at 1 month (*P* = 0.037).

**Table 2.** Top-Down postoperative outcome measures at 1, 3, 6, and 12 months

Variable	Value	*P*
Number of patients 1 month 3 months 6 months 12 months	60 60 59 57	
IPSS, median (range) 1 month 3 months 6 months 12 months	5 (0–19) 4 (0–21) 2 (0–14) 2 (0–9)	0.045
QoL, median (range) 1 month 3 months 6 months 12 months	1 (0–5) 1 (0–5) 0 (0–4) 0 (0–4)	0.04
PVR, median (range) 1 month 3 months 6 months 12 months	36.5 (0–194) 55 (0–190) 26 (0–142) 29 (0–153)	0.12
Q_max_, mL/s, median (range) 1 month 3 months 6 months 12 months	23.6 (13–48.7) 25.4 (12.3–48.7) 29.4 (10.8–48) 28.5 (9.6–58.6)	0.17
PSA level, ng/mL, median (range) Preoperative 3-months	6 (0.84–27.1) 0.59 (0.16–9)	<0.001

The median (range) percentage of IPSS improvement at 6 and 12 months was 84 (44–94.3)% and 88.9 (42.2–100)%, respectively. Patients reported an improvement in QoL by a median (range) percentage of 66.7 (0–100)% at 6 months. At 12 months, patients reported a median (range) percentage of improvement in QoL of 81.7 (33.3–100)%. At 6 and 12 months, the median (range) percentage of Q_max_ improvement was 218.2 (116–608)% and 209.8 (100–684)%, respectively. The PVR decreased by a median (range) percentage of 90.6 (26.2–100)% at 6 months and 96.4 (36.1–100)% at 12 months.

### Learning curve assessment

There were no significant differences among the compared groups in terms of the operative and postoperative outcomes (Table 3). However, when we compared the first and last groups, we found significant difference in terms of percentages of PVR and Q_max_ improvements (*P* = 0.048 and *P* = 0.032, respectively).

**Table 3.** Comparison among learning curve groups in relation to the operative and postoperative outcomes

Variable	First 20 cases	Second 20 cases	Last 20 cases	*P*
Enucleation time, min, median (range)	91 (50–130)	86 (25–184)	67 (45–200)	0.56
Resected weight, g, median (range)	95 (27–190)	81 (44–236)	86 (34–242)	0.76
Enucleation efficiency, g/min, median (range)	0.86 (0.4–1.34)	0.9 (0.42–1.9)	0.91 (0.63–3.7)	0.99
SUI, *n* (%)	1 (5)	2 (10)	0	0.35
12-month % IPSS improvement, median (range)	81.25 (72.7–93.3)	82.54 (42.2–100)	82.35 (60–100)	0.98
12-month % QoL improvement, median (range)	80 (50–100)	81.7 (33.3–100)	85.5 (66.7–100)	0.58
12-month % PVR improvement, median (range)	93.3 (36.1–100)	90.86 (58.3–100)	100 (43.8–100)	0.28
12-month % Q_max_ improvement, median (range)	170.3 (100–198.2)	200.4 (103–436.5)	235.3 (110–648)	0.2

## Discussion

In the last decade, HoLEP has gained popularity as a strong alternative to simple prostatectomy for the management of large prostatic adenomas. HoLEP has better outcomes than traditional TURP and less morbidity than open prostatectomy [12,13]. However, the major drawback for the widespread application of HoLEP is the relatively long learning curve required to effectively master the technique. Traditional HoLEP has undergone some modifications to simplify the procedure and to shorten its operative time [7,8,14,15].

In 2017, York et al. [10] introduced the Top-Down technique, as a modification of classic HoLEP, aiming to shorten the operative time and lessen the number of procedures required to master the technique. Another advantage of the Top-Down HoLEP is a reduced risk of overstretching the sphincter when cutting the mucosal flap attached to the sphincter. In our opinion, the Top-Down technique has many advantages, including easier division of the mucosal strip during the distal apical extent of the lateral lobe dissection. It also eliminates the need for the encircling technique. We previously presented the 3-month outcomes of Top-Down HoLEP [11]. In the present study, we evaluate the 12-month outcomes of the Top-Down technique in terms of the operative data and postoperative outcomes.

The mean enucleation time reported in the literature varies from 36 to 140 min [7,8,14]. In our present study, the median (range) enucleation time was 80 (25–200) min, whereas York et al. [10] reported a mean enucleation time of 43.8 min. York et al. [10] also reported a mean enucleated volume of 74.5 g in comparison to a median of 90 g in our present cohort. In our present study, the median (range) enucleation efficiency was 0.9 (0.4–3.7) g/min, which was similar to that reported by York et al. [10]. York et al. [10] reported that both enucleation time and rate were faster with the novel Top-Down approach [16].

Enucleation time depends on many factors such as prostate size and the surgeon’s experience. However, the mean prostatic sizes of these studies were less than the median prostate size in our present cohort. We noticed that performing apical dissection from top-down resulted in easy visualisation of the mucosal strip. This approach eliminates the need to encircle the mucosal strip, hence reducing enucleation time. This may confirm the theory that the Top-Down technique lessens operative time. However, more studies are warranted to confirm this theory.

In our present cohort, no intraoperative complications were recorded, apart from a single patient with simple bladder mucosal laceration. Minagawa et al. [8] did not report any intraoperative complications in their cohort consisting of 26 patients using the *en bloc* anteroposterior HoLEP technique. In another study, Elzayat et al. [17] reported that 12/552 (2.2%) patients had intraoperative complications. This exemplifies the safety profile of the new HoLEP technique when compared with the classic approach.

One of the major differences between TURP and HoLEP is the short catheterisation time [13]. In the literature, the post-HoLEP catheterisation time is 1–2 days [17,18]. In our present study, most patients had their catheters removed before 24-h postoperatively, with a failed TOV in only two patients (3.3%). Our present results confirm the possibility and safety of shortening the post-HoLEP catheterisation time. One of the present patients developed clot retention (1.6%) due to returning to manual work early. In another cohort, four patients (0.7%) developed clot retention after classic HoLEP and were readmitted within 4-weeks postoperatively [17]. This confirms the similarity of the low bleeding probability in both techniques.

SUI is a major complication after HoLEP. Many studies have postulated the mechanism of SUI; however, it is believed to be multifactorial. Risk factors include old age, diabetes mellitus, muscular asthenia, prolonged catheterisation, prostate size, operative time, operative technique, and impaired mental status [19–21]. In the present study, three patients had SUI at the 1-month follow-up. However, only one patient had persistent SUI at his last follow-up visit at 12 months. In their retrospective cohort, Lerner et al. [19] found that 17 patients (26%) had SUI at the 3-month follow-up and only two patients (3%) remained incontinent at 12 months. Lerner et al. [19] state that the long duration between cases (>7 weeks) was the only predictor of SUI at 3 months for their patients. These factors could explain their difference with our cohort. In another study, Endo et al. [7] described a significantly higher rate of SUI with the classic HoLEP when compared with the anteroposterior HoLEP (25.2% vs 2.7%). York et al. [16] reported better continence rate with the Top-Down approach compared to the conventional approach. In our opinion, the Top-Down technique is associated with less overstretch of the sphincter than classic HoLEP.

In the present study, there were significant differences between the IPSS and QoL at the 1-, 3-, 6- and 12-month follow-ups. Moreover, there was a significant improvement when comparing Q_max_ at the 1- and 12-month follow-ups. Hurle et al. [22] had similar results, with significant improvement for all follow-up parameters.

In their study, Elshal et al. [23] found that the enucleation time and efficiency started plateauing after the first 40 procedures. In our present cohort, there were no significant differences in the enucleation time and efficiency among learning groups. This can probably be explained by the fact that in our present cohort all the procedures were peformed by a single surgeon familiar with HoLEP, while in the Elshal et al. [23] study HoLEP was performed by three surgeons, two of them were familiar with TURP and started doing supervised HoLEP procedures.

Our present study has some limitations including the retrospective nature of the study. We attempted to overcome this limitation by the prospective inclusion of patients that were operated on by the same surgeon. Moreover, the study has a relatively small sample size. The study represents an initial report for a new technique and the sample size is comparable to those of other studies [7,19]. Lastly, despite the fact that this study evaluated the learning curve of the described technique, further studies are required to describe the learning of the Top-Down technique for HoLEP beginners.

## Conclusion

Our present results confirm that the Top-Down technique is satisfactory in terms of the operating time and SUI rate after HoLEP. Our results show that the Top-Down approach can be learned rapidly by urologists who are familiar with classic HoLEP. Further comparative studies are required to determine the effect of this technique on HoLEP’s steep learning curve for HoLEP beginners.
